# Two new species of *Coniopteryx* Curtis from China (Neuroptera, Coniopterygidae)

**DOI:** 10.3897/zookeys.1015.57451

**Published:** 2021-02-10

**Authors:** Yaru Zhao, Davide Badano, Zhiqi Liu

**Affiliations:** 1 Department of Entomology, China Agricultural University, Beijing, 100094, China China Agricultural University Beijing China; 2 Department of Biology and Biotechnologies ‘Charles Darwin’, Sapienza University of Rome, Piazzale A. Moro 500185, Rome, Italy Sapienza University of Rome Rome Italy

**Keywords:** Dustywings, faunistics, identification key, lacewings, morphology, taxonomy

## Abstract

Two new species of Coniopterygidae, Coniopteryx (Coniopteryx) tenuisetosa**sp. nov.**, and Coniopteryx (Coniopteryx) serrata**sp. nov.**, are described from China. Both species differ from congeners in characters of the male genitalia. Coniopteryx (Coniopteryx) alticola Sziráki, 2002, is recorded from China for the first time. A key to species of the genus *Coniopteryx* from China is presented.

## Introduction

Coniopterygidae, or dustywings – after the wax covering their bodies – are one of the most diverse lineages of Neuroptera, including 571 known species (Oswald and Machado 2018). Coniopterygids are common and often abundant in woody environments worldwide, though they are easily overlooked due to their small size, being the dwarfs among lacewings. Nevertheless, dustywings are of major phylogenetic interest, as they are the sister group to all the other Neuroptera, diverging from them in the Permian (Winterton et al. 2018; Vasilikopoulos et al. 2020). Their evolutionary history has been characterized by miniaturization, with a reduction of their overall body size, with major impacts on their morphology and anatomy (Randolf et al. 2017; Randolf and Zimmermann 2019). Like most lacewings, dustywings are predators both as larvae and adults, feeding on small arthropods such as mites, scale insects and aphids (Pantaleoni 2007). Coniopterygidae are divided in three subfamilies, Brucheiserinae, Aleuropteryginae and Coniopteryginae, of which the last group is the richest in species (Oswald and Machado 2018; Handschuh and Aspöck 2020). The genus *Coniopteryx*Curtis (1834) is in turn the most diverse group of Coniopteryginae attaining a sub-cosmopolitan distribution (Meinander 1972; Sziráki 2011). Meinander (1972) divided this genus into six subgenera based on morphology of genitalia: *Coniopteryx* s. str. (77 spp.), *Xeroconiopteryx* Meinander, 1972 (85 spp.), *Protoconiopteryx* Meinander, 1972 (1 sp.) *Scotoconiopteryx* Meinander, 1972 (33 spp.), *Holoconiopteryx* Meinander, 1972 (8 spp.), and *Metaconiopteryx* Meinander, 1972 (4 spp.). Eleven additional species are not presently allocated to a subgenus (see also Meinander 1990; Sziráki 2011, 2015, Martins and Amorim 2016). Twenty-six species of *Coniopteryx* are known for China, belonging to the subgenera *Coniopteryx* (22 spp.) and *Xeroconiopteryx* (4 spp.). This paper describes two new species of *Coniopteryx* s. str. from China. We also report for the first time the presence of *Coniopteryx alticola*Sziráki 2002 in China, increasing the number of *Coniopteryx* species known from this country to 29.

## Material and methods

Examined specimens are deposited in the Entomological Museum of China Agricultural University, Beijing (**CAU**), which are preserved in 95% ethyl alcohol. The abdomen was dissected from the body and macerated in a heated solution of 5% KOH for 5 minutes, then rinsed in water and 95% ethyl ethanol. And finally, the cleared abdomen was transferred to glycerol for dissection and study. After examination, the abdomen was preserved in glycerol and stored in a microtube. The head and the thorax of the specimen were preserved in 95% ethyl alcohol and stored in another microtube. Morphological terminology mostly follows Meinander (1972), Aspöck and Aspöck (2008) and Handschuh and Aspöck (2020). Specimens were examined with an Optec SZ760 stereomicroscope. Photos were taken with a Nikon D5300 digital camera attached to a Leica DM2500 stereomicroscope. The resulting images were edited and processed with Adobe Photoshop CC 2018.

## Taxonomy

### Family Coniopterygidae Burmeister, 1839

#### Subfamily Coniopteryginae Burmeister, 1839

**Genus *Coniopteryx* Curtis, 1834**

**Subgenus *Coniopteryx* (s. str.) Curtis, 1834"**

**Type species.***Coniopteryx tineiformis* Curtis, 1834.

**Diagnosis.** Male genitalia: gonocoxites 9 and sternite 9 as distinct sclerites; gonocoxites 9 divided into a pair of lateral sclerites; sternite 9 about as broad as high in lateral view, with a prominent lateral process, forming a dorso-caudal angle, median apical incision present; gonapophyses 10 generally sclerotized (Meinander 1972; Sziráki 2011; Handschuh and Aspöck 2020).

### Key to the species of *Coniopteryx* from China (males)

Note: Coniopteryx (Coniopteryx) abdominalis Okamoto, 1905 is not included in the key as the specimen is unavailable for study.

**Table:** 

1	Apical part (stylus) arising well before the caudal end of basal part (gonarcus) in gonocoxites 9 (Fig. 1a–c) subgenus Xeroconiopteryx	**2**
–	Apical part (stylus) arising from the caudal end of basal part (gonarcus) in gonocoxites 9 (Figs 1d, 6a, b, 8a, b, 10a, b) subgenus Coniopteryx	**5**
2	Anterior margin arched on sternite 9 laterally (Fig. 1a)	**C. (X.) mongolica**
–	Anterior margin straight on sternite 9 laterally (Fig. 1b, c)	**3**
3	Apodeme along anterior margin ventrally incomplete (Fig. 2a)	**C. (X.) qiongana**
–	Apodeme along anterior margin ventrally complete (Fig. 2b)	**4**
4	Apical part (stylus) of gonocoxites 9 slender laterally (Fig. 1b)	**C. (X.) minana**
–	Apical part (stylus) of gonocoxites 9 widening in middle part laterally (Fig. 1c)	**C. (X.) unguigonarcuata**
5	Male head with prominent frontal lobe (Fig. 5a–c) *Coniopteryx lobifrons* group (3 species)	**6**
–	Male head without prominent frontal lobe (Figs 7b, 9b)	**8**
6	Distal part of gonocoxites 10 hammer-like laterally (Fig. 3a)	**C. (C.) dactylifrons**
–	Distal part of gonocoxites 10 not hammer-like laterally (Figs 3b, 6a, b)	**9**
7	Gonocoxites 10 subtriangular apically laterally (Fig. 3b)	**C. (C.) protrufrons**
–	Gonocoxites 10 not subtriangular apically laterally (Fig. 6a, b)	**C. (C.) alticola**
8	Male antennae with peculiar outgrowths (Fig. 4a–d) *Coniopteryx falciger* group (4 species)	**9**
–	Male antennae without peculiar outgrowths (Figs 7a, b, 9a, b)	**12**
9	The first two flagellar segments with acute projections (Fig. 4a)	**C. (C.) bispinalis**
–	The first two flagellar segments without acute projections (Fig. 4b–d)	**10**
10	The last flagellar segments with a curved claw-like hair (Fig. 4b)	**C. (C.) prehensilis**
–	The last flagellar segments without claw-like hairs (Fig. 4c, d)	**11**
11	Antennae with one long bristle on middle segments (Fig. 4c)	**C. (C.) unispinalis**
–	Antennae with two acute projections on middle segments (Fig. 4d)	**C. (C.) gibberosa**
12	Distal part of gonocoxites 10 pick-like (Fig. 10a, b, g) or hammer-like in shape (Fig. 3c, d) *Coniopteryx tineiformis* group (4 species)	**13**
–	Distal part of gonocoxites 10 not pick- and hammer-like in shape (Figs 3f–h, 8a, b) *Coniopteryx exigua* group (13 species)	**16**
13	Bottom of median incision rounded in a U-shape (Fig. 2c)	**C. (C.) wuyishana**
–	Bottom of median incision narrowing in a V-shape (Fig. 10e, f)	**14**
14	Processus apicalis of gonocoxites 10 pick-like (Fig. 10a, b)	**C. (C.) serrata sp. nov.**
–	Processus apicalis of gonocoxites 10 hammer-like (Fig. 3c, d)	**15**
15	Median incision deep in ventral view (Fig. 2d)	**C. (C.) alifera**
–	Median incision shallow in ventral view (Fig. 2e)	**C. (C.) pygmaea**
16	Anterior margin arched on sternite 9 laterally (Fig. 1d)	**C. (C.) praecisa**
–	Anterior margin straight on sternite 9 laterally (Fig. 8a, b)	**17**
17	Distal part of gonocoxites 10 sickle-like in shape (Fig. 3e)	**C. (C.) crispicornis**
–	Distal part of gonocoxites 10 not sickle-like in shape (Figs 3f–h, 8a, b)	**18**
18	Basal flagellar segments more than three times as long as wide (Fig. 4e)	**C. (C.) miraparameris**
–	Basal flagellar segments at most two times as long as wide (Fig. 7a, b)	**19**
19	Distal part of gonocoxites 10 widening abruptly (Meinander 1972: 245, fig. 156)	**C. (C.) pallescens**
–	Distal part of gonocoxites 10 not widening abruptly (Figs 3f–h, 8g)	**20**
20	Caudal edge of gonocoxites 10 serrate apically (Fig. 8g)	**C. (C.) tenuisetosa sp. nov.**
–	Caudal edge of gonocoxites 10 not serrate apically (Fig. 3f–h)	**21**
21	Distal part of gonocoxites 10 directed downwards perpendicularly (Fig. 3f)	**C. (C.) aspoecki**
–	Distal part of gonocoxites 10 not directed downwards perpendicularly (Fig. 3g, h)	**22**
22	Middle part of gonocoxites 10 curved downward in a blunt angle (Fig. 3g)	**23**
–	Middle part of gonocoxites 10 not curved downward (Fig. 3h)	**24**
23	Median incision U-shaped (Meinander 1972: 244, fig. 155)	**C. (C.) sularis**
–	Median incision V-shaped (Fig. 2f)	**C. (C.) choui**
24	Sternite 9 with strong longitudinal apodeme (Fig. 2g)	**C. (C.) plagiotropa**
–	Sternite 9 without longitudinal apodeme (Fig. 2h–j)	**25**
25	Median incision almost equal to the half of width of sternite 9 (Fig. 2h)	**26**
–	Median incision smaller than the half of width of sternite 9 (Fig. 2i, j)	**27**
26	Median incision very deep and narrow (Fig. 2h)	**C. (C.) compressa**
–	Median incision very shallow and wide (Meinander 1972: 238, fig. 151)	**C. (C.) ambigua**
27	Median incision without a transverse inner plate in caudal view (Fig. 1e)	**C. (C.) exigua**
–	Median incision with a transverse inner plate in caudal view (Fig. 1f)	**C. (C.) guangxiana**

**Figure 1.**
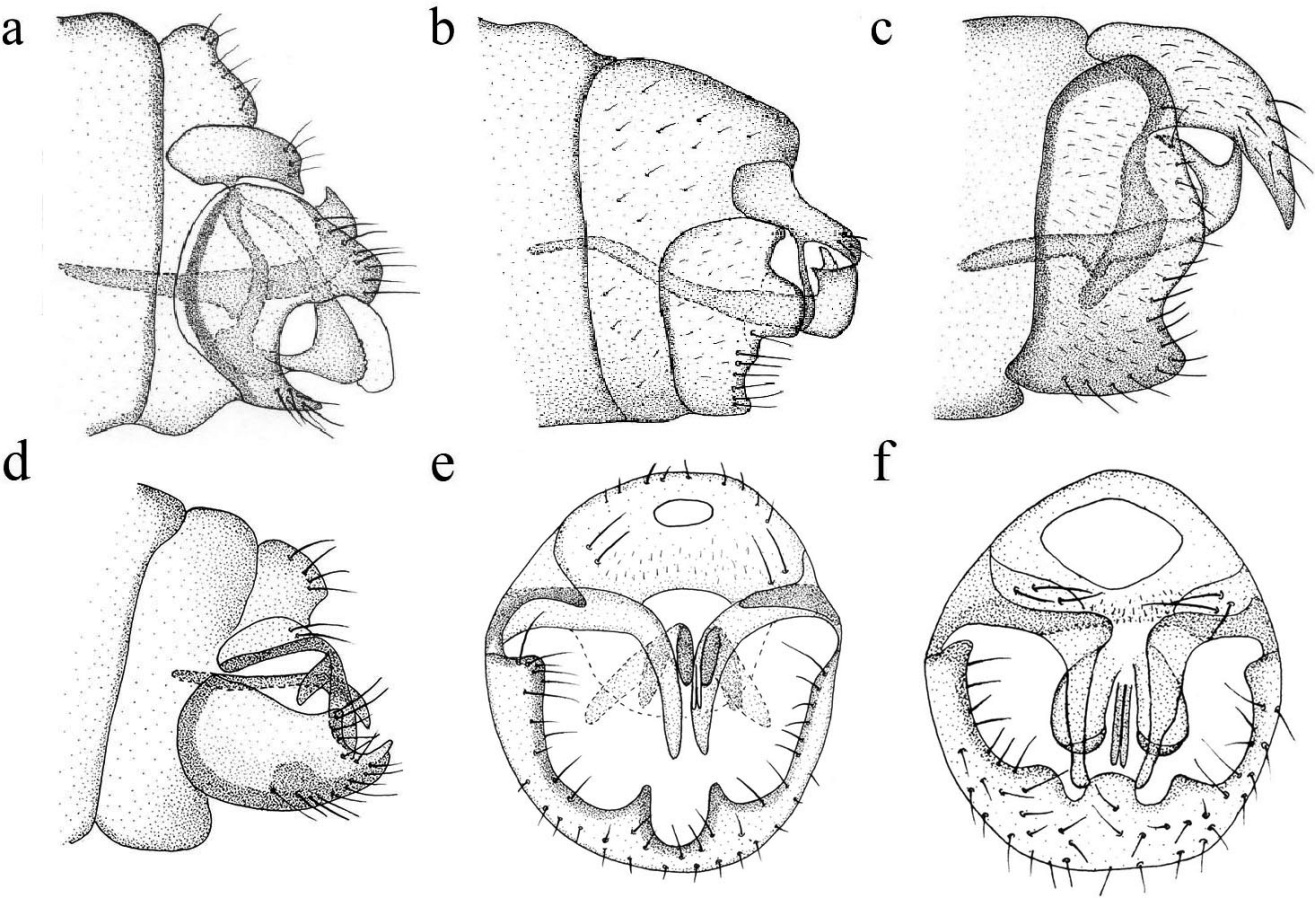
Genitalia of *Coniopteryx* species **a***C. mongolica* (lateral view) **b***C. minana* (lateral view) **c***C. unguigonarcuata* (lateral view) **d***C. praecisa* (lateral view) **e***C. exigua* (caudal view) **f***C. guangxiana* (caudal view).

**Figure 2.**
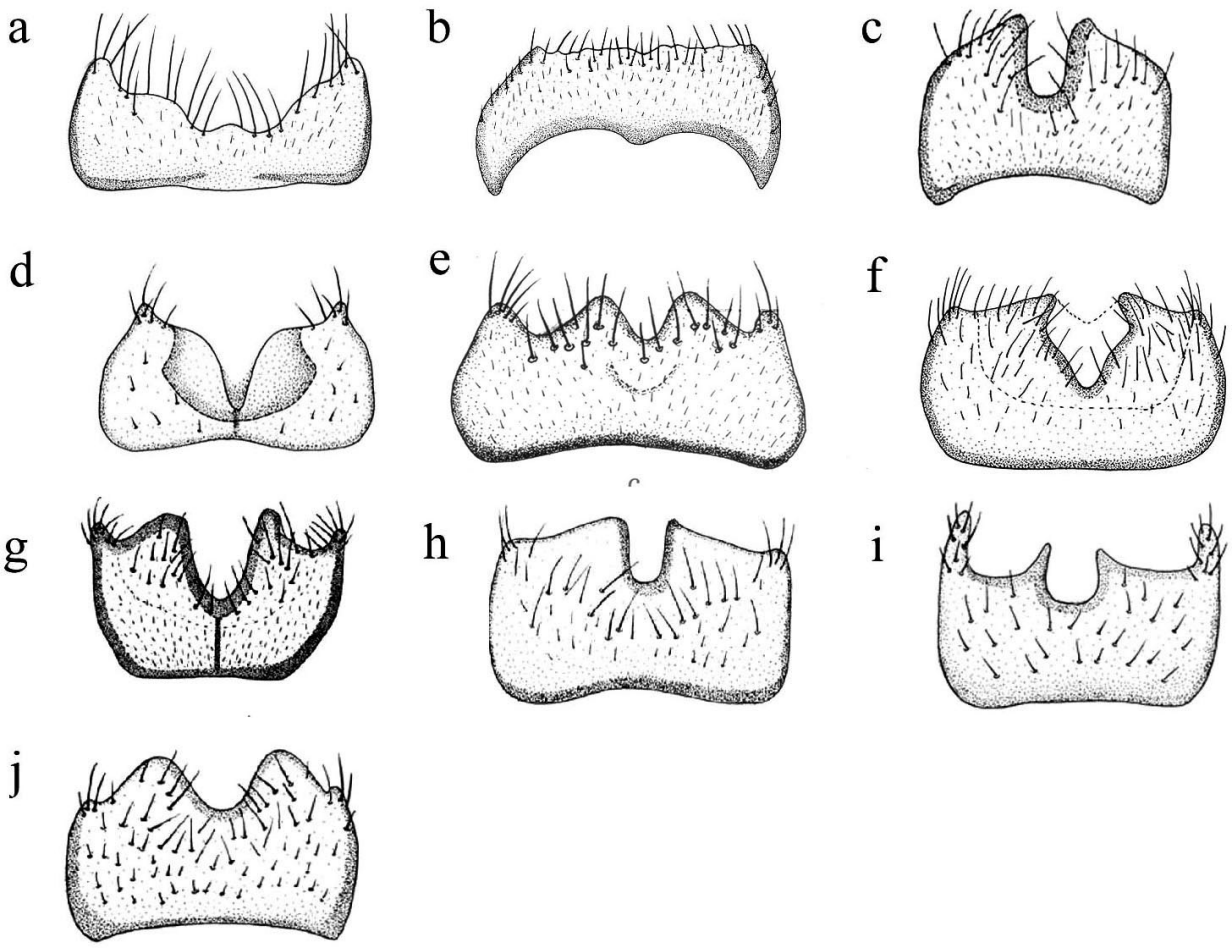
Sternite 9 of *Coniopteryx* species, ventral view **a***C. qiongana***b***C. unguigonarcuata***c***C. wuyishana***d***C. alifera***e***C. pygmaea***f***C. choui***g***C. plagiotropa***h***C. compressa***i***C. exigua***j***C. guangxiana*.

**Figure 3.**
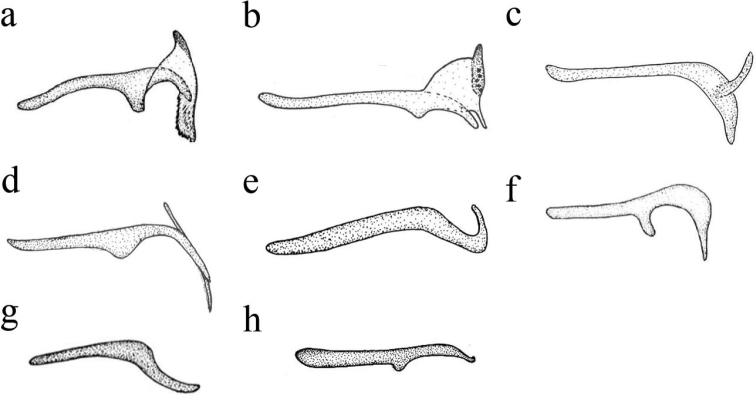
Gonocoxites 10 of *Coniopteryx* species, lateral view **a***C. dactylirons***b***C. protrufrons***c***C. alifera***d***C. pygmaea***e***C. crispicornis***f***C. aspoecki***g***C. choui***h***C. plagiotropa*.

**Figure 4.**
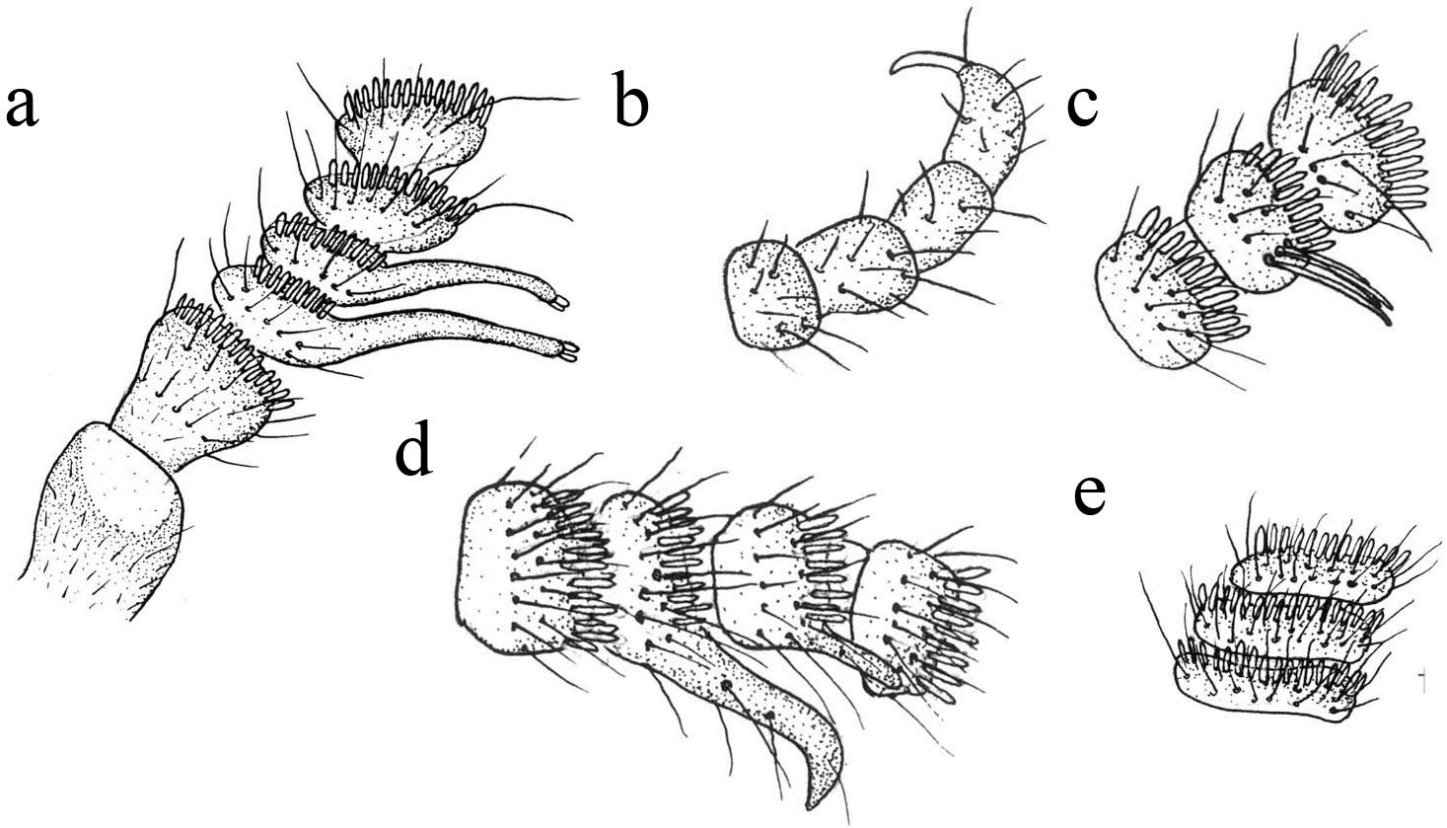
Antennae of *Coniopteryx* species **a***C. bispinalis* (antennal segments 1–6) **b***C. prehensilis* (distal part of antennal segments) **c***C. unispinalis* (antennal segments 11–13) **d***C. gibberosa* (antennal segments 8–11) **e***C. miraparameris* (antennal segments 8–10).

#### Coniopteryx (Coniopteryx) alticola

Taxon classificationAnimaliaNeuropteraConiopterygidae

Sziráki, 2002

2EF46DB5-18E3-57DA-8793-05FBCB5D4277

Figs 5 , 6

##### Material examined.

1 male, China: Yunnan (Province): Puer (City): Meizihu Park, [22.7551°N, 100.9845°E], 20.iii.2019, leg. Yaru Zhao. 3 males, China: Yunnan (Province): Yuanjiang (County): Jiangdong Park, [23.6001°N, 102.0098°E], 18.iii.2019, leg. Yaru Zhao (CAU).

**Figure 5.**
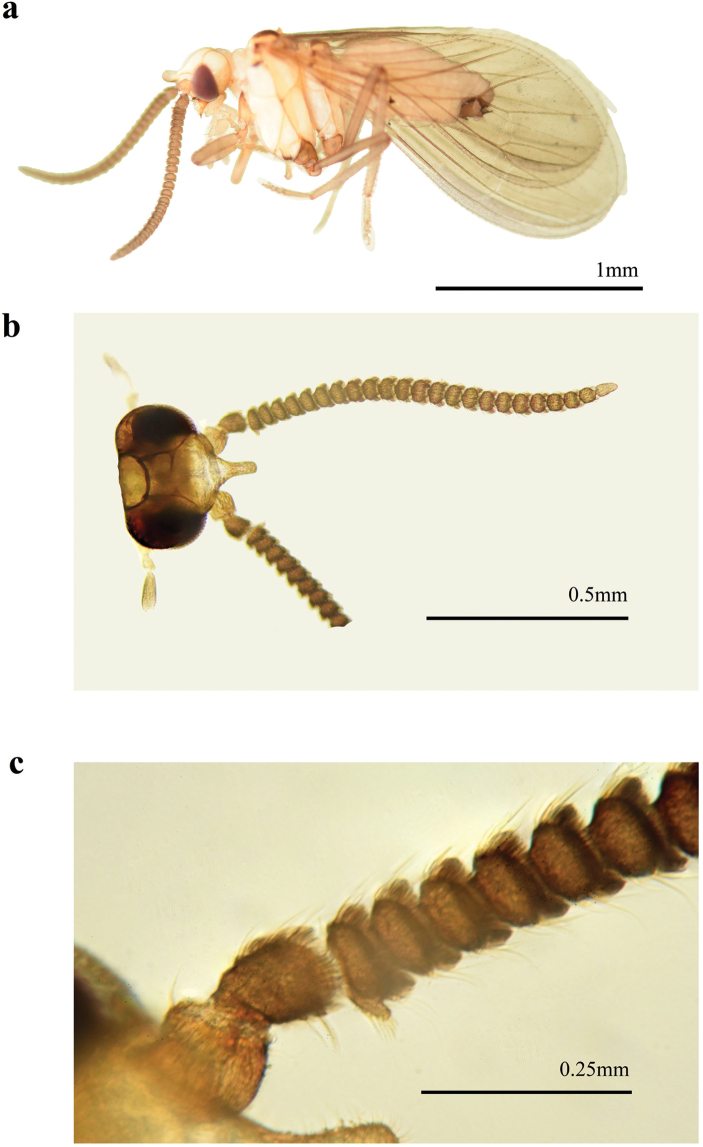
Coniopteryx (Coniopteryx) alticola Sziráki, 2002, male **a** habitus, lateral view **b** head, dorsal view **c** male, first flagellomere, dorsal view.

**Figure 6.**
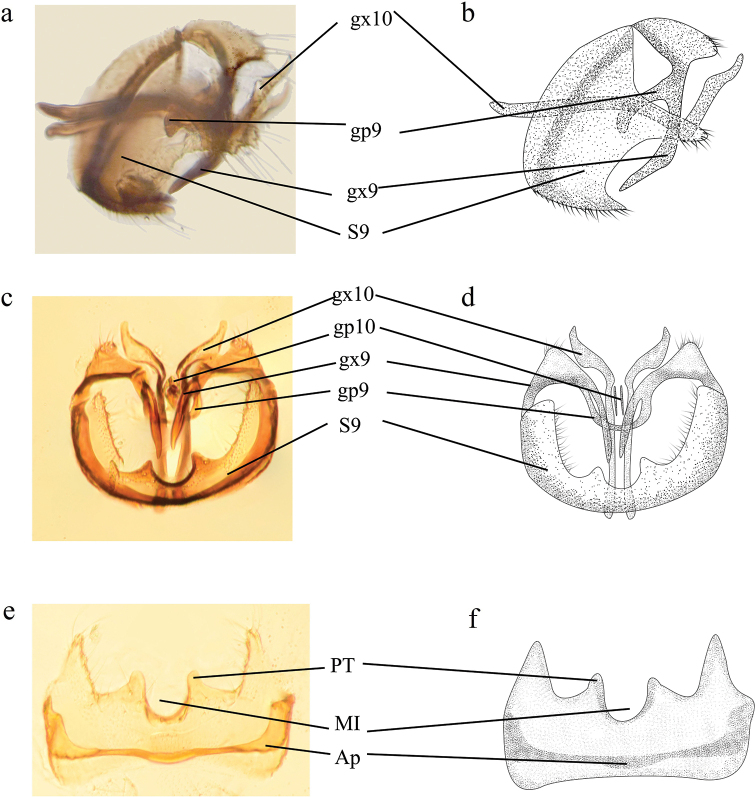
Coniopteryx (Coniopteryx) alticola Sziráki, 2002, male genitalia **a, b** genitalia, lateral view **c, d** genitalia, caudal view **e, f** Sternite 9 (S9), ventral view.

##### Measurements.

Forewing length 1.7 mm, width 0.9 mm. Hindwing length 1.4 mm, width 0.6 mm.

##### Redescription.

**Male: *Head*** (Fig. 5a–c). Frons with prominent anterior process. Antennae brown, 25-segmented, 1.0 mm in length. Basal flagellomeres two times as long as broad. Subsequent flagellomeres tapering gradually. Apical flagellomere almost as long as wide.

***Thorax*.** Light brown. Meso- and metanotum with dorsal dark spots. Legs yellowish brown.

***Wing*.** Wing membrane light greyish brown, almost hyaline.

***Male terminalia*** (Fig. 6a–f). Accord with the description by Sziráki (2002).

##### Remarks.

Coniopteryx (Coniopteryx) alticola Sziráki, 2002 belongs to the *C. lobifrons* species group (Sziráki 2004). The members of this group are characterized by the presence of a prominent process on the frons and of a protuberance on the first flagellomere (Fig. 5b, c). Coniopteryx (C.) alticola was originally described from Thailand (Sziráki 2002) and the examined specimens represent the first record of this species from China.

##### Distribution.

China, Yunnan, first record; Thailand.

#### Coniopteryx (Coniopteryx) tenuisetosa sp. nov.

Taxon classificationAnimaliaNeuropteraConiopterygidae

54DE99B4-3D62-5510-BB9B-574663ED7A60

http://zoobank.org/95D212F4-D6D2-4F6C-8A1E-FC7C7073F128

Figs 7 , 8

##### Type material.

***Holotype*** 1 male, China: Tibet (Province): Linzhi (City), [29.6019°N, 94.4168°E], 8.vi.2019, leg. Yaru Zhao (CAU). ***Paratypes*** 39 males and 54 females, same data as holotype (CAU).

##### Other material.

2 males, China: Yunnan (Province): Lincang (City): Fengqing (County), [24.5934°N, 99.9001°E], 23.iv.1981, leg. Chikun Yang (CAU). 1 male, China: Yunnan (Province): Baoshan (City): Tengchong (County), [25.0199°N, 98.4800°E], 25.iv.1981, leg. Chikun Yang (CAU). 1 male, China: Yunnan (Province): Ruili (County): Mengxiu (Township), [25.0667°N, 98.4167°E], 2.v.1981, leg. Chikun Yang (CAU). 3 males, China: Yunnan (Province): Ruili (County): Mengxiu (Township): Nanjingli (Village), [24.0917°N, 97.8460°E], 2.v.1981, leg. Fasheng Li (CAU). 5 males, China: Tibet (Province): Linzhi (City): Linzhi (County): Gengzhang (Township), [29.7298°N, 94.0870°E], 1.vi.1978, leg. Fasheng Li (CAU). 1 male, China: Tibet (Province): Linzhi (City): Linzhi (County), [29.6019°N, 94.4168°E], 3.vi.1978, leg. Fasheng Li (CAU). 1 male, China: Tibet (Province): Linzhi (City): Bomi (County): Yigong (Township), [30.2389°N, 94.8523°E], 28.vi.1978, leg. Fasheng Li (CAU). 2 males, China: Tibet (Province): Linzhi (City): Bomi (County): Zhamu (Township), [29.7103°N, 95.5857°E], 1.vii.1978, leg. Fasheng Li (CAU). 1 male, China: Tibet (Province): Linzhi (City): Milin (County), [29.0428°N, 93.8898°E], 4.vi.1978, leg. Fasheng Li (CAU). 1 male, China: Tibet (Province): Linzhi (City): Lulang (County), [29.8208°N, 94.7382°E], 2.viii.1978, leg. Fasheng Li (CAU). 2 males, China: Tibet (Province): Linzhi (City): Chayu (County), [29.7103°N, 95.5857°E], 2.viii.1978, leg. Fasheng Li (CAU). 7 males, China: Tibet (Province): Linzhi (City): Milin (County), [29.0423°N, 94.2364°E], 9.vi.2019, leg. Yaru Zhao (CAU).

##### Diagnosis.

Male genitalia: median apical incision shallow, U-shaped, less than half of sternite 9 length; terminal process blunt in lateral view; distal part of gonocoxites 10 short and stout, with tiny hairs.

##### Measurements.

Forewing length 2.0–2.8 mm, width 1.0–1.3 mm. Hindwing length 1.5–1.7 mm, width 0.5–0.7 mm.

##### Description.

**Figure 7.**
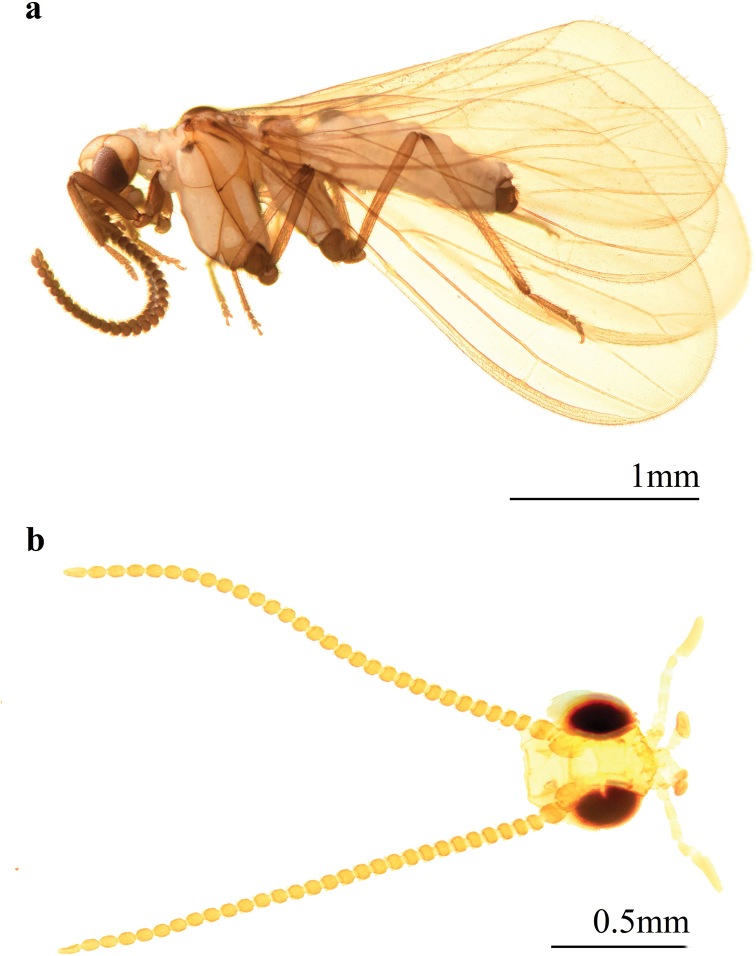
Coniopteryx (Coniopteryx) tenuisetosa sp. nov., male **a** habitus, lateral view **b** head, dorsal view.

**Male: *Head*** (Fig. 7a, b). Brown. Frons without projections. Compound eyes large. Antennae brown, 28-segmented, 1.2–1.5 mm in length. Scape and pedicel broad and blunt. Basal flagellomeres wider than long, distal flagellomeres gradually tapering toward apex, apical flagellomere almost as long as wide. Apices of flagellomeres covered with scattered scale-like hairs and two whorls of setae. Maxillary and labial palps brown.

***Thorax*.** Yellowish brown. Meso- and metanotum dorsal dark spots. Legs yellowish brown, except the brown coxae.

***Wing*.** Wing membrane light greyish brown, almost hyaline.

**Figure 8.**
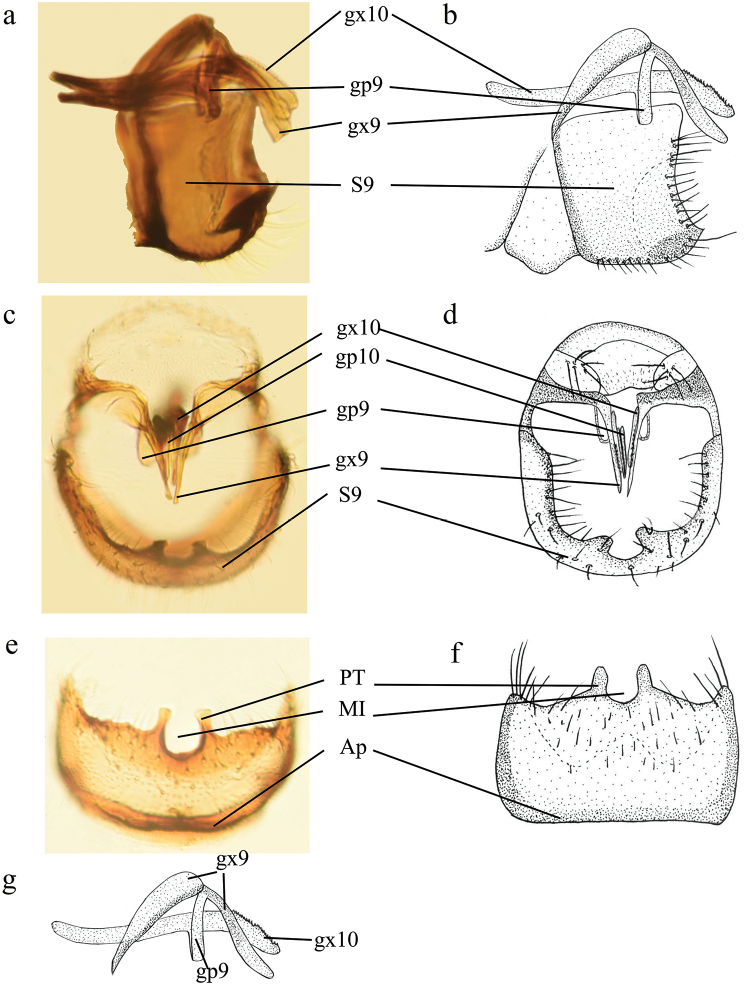
Coniopteryx (Coniopteryx) tenuisetosa sp. nov., male genitalia **a, b** genitalia, lateral view **c, d** genitalia, caudal view **e, f** sternite 9, ventral view **g** gonocoxites 10 (gx10), gonocoxites 9 (gx9) and gonapophyses 9 (gp9), lateral view.

***Male terminalia*** (Fig. 8a–g). Sternite 9 higher than wide in lateral view; anterior margin straight laterally; ventral apodeme along anterior margin not interrupted; lateral process rounded and blunt; terminal process short and acute in lateral view, rounded and blunt in caudal view; median apical incision shallow and U-shaped, and its depth less than half the length of the sternite 9. Gonocoxites 10 long and slender, bent downwards near apex, distal portion serrated and covered with many tiny setae. Gonapophyses 10 as a pair of long, slender rods.

##### Distribution.

China (Tibet, Yunnan).

##### Etymology.

The species name *tenuisetosa* “thin-haired” is a composed adjective of Latin derivation, referring to the thin setae on the distal portion of gonocoxites 10.

##### Remarks.

The new species is similar to Coniopteryx (Coniopteryx) aspoecki Kis, 1967, but the two species differ in configuration of the male genitalia. In particular, Coniopteryx (Coniopteryx) tenuisetosa is characterized by a short, not prominent terminal process of sternite 9 in lateral view, while it is prominent and arched in *C. aspoecki*. Moreover, in the new species, the distal portion of gonocoxites 10 is relatively robust and serrated, while in *C. aspoecki* it is thin, apically tapered and smooth.

#### Coniopteryx (Coniopteryx) serrata sp. nov.

Taxon classificationAnimaliaNeuropteraConiopterygidae

8E1E2B2A-973B-501E-A5EC-E3A0D8488249

http://zoobank.org/5779FE7C-048C-49D6-9218-88C254002379

Figs 9 , 10

##### Type material.

***Holotype*** 1 male, China: Yunnan (Province): Puer (City): Meizihu Park, [22.7551°N, 100.9845°E], 20.iii.2019, leg. Yaru Zhao. ***Paratype*** 1 male, same data as holotype (CAU).

##### Other material.

1 male, China: Yunnan (Province): Ruili (County): Mengxiu (Township), [25.0667°N, 98.4167°E], 2.v.1981, leg. Chikun Yang (CAU). 1 male, China: Yunnan (Province): Puer (City): Simao (District), [22.7860°N, 100.9798°E], 7.vi.1981, leg. Chikun Yang (CAU). 3 males, China: Yunnan (Province): Ruili (County): Mengxiu (Township): Tuanjiezhai (Village), [24.0917°N, 97.8460°E], 30.iii.2019, leg. Yaru Zhao (CAU).

##### Diagnosis.

Male genitalia: median apical incision V-shaped. Its depth is more than the half of the length of sternum 9. Terminal process long and acute in lateral view. Distal part of gonocoxites 10 bent upwards perpendicularly.

##### Measurements.

Forewing length 2.2–2.4 mm, width 0.8–1.1 mm. Hindwing length 1.5–1.8 mm, width 0.7–0.8 mm.

##### Description.

**Figure 9.**
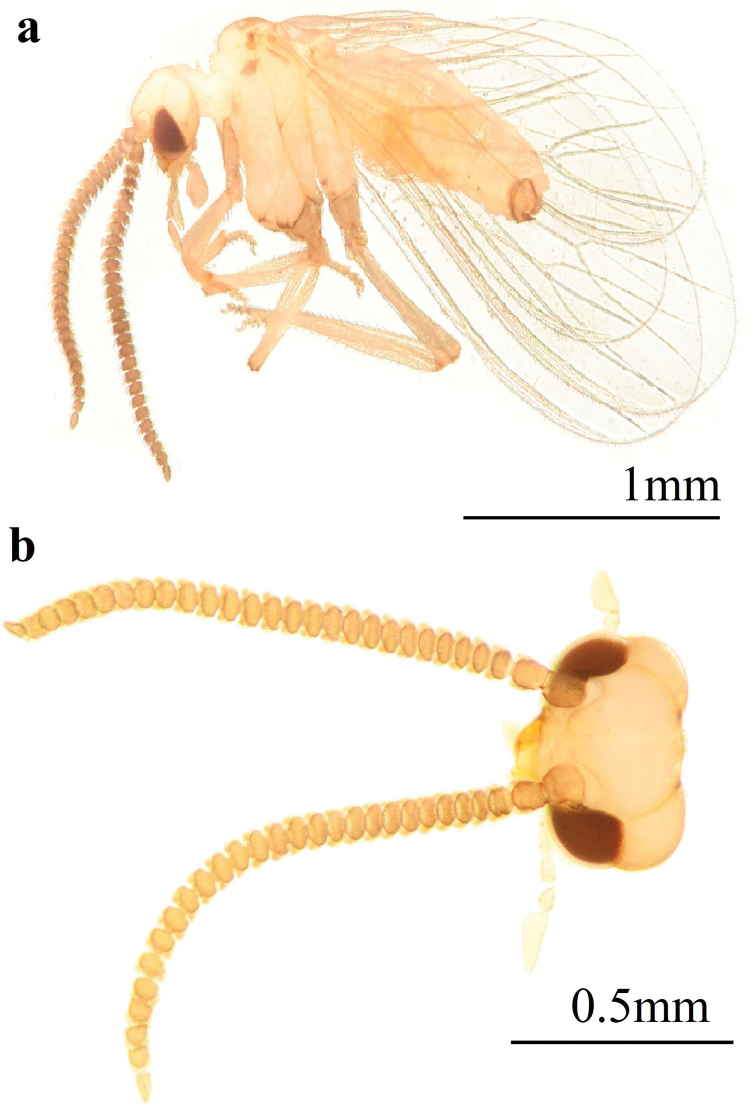
Coniopteryx (Coniopteryx) serrata sp. nov., male **a** habitus, lateral view **b** head, dorsal view.

**Male: *Head*** (Fig. 9a, b). Yellowish brown. Frons without projections. Compound eyes large. Antennae brown, 27–28-segmented, 1.2 mm in length. Scape and pedicel long and narrow. Basal flagellomeres two times wider than long, apical flagellomeres tapered. Flagellomeres scattered with scale-like setae at apex and two circles of hair-like sensilla; setae present on most segments except basal ones. Maxillary and labial palps yellowish brown.

***Thorax*.** Brown. Meso- and metanotum with dorsal dark spots. Legs yellowish brown except the brown coxae.

***Wing*.** Wing membrane light greyish brown, almost hyaline.

**Figure 10.**
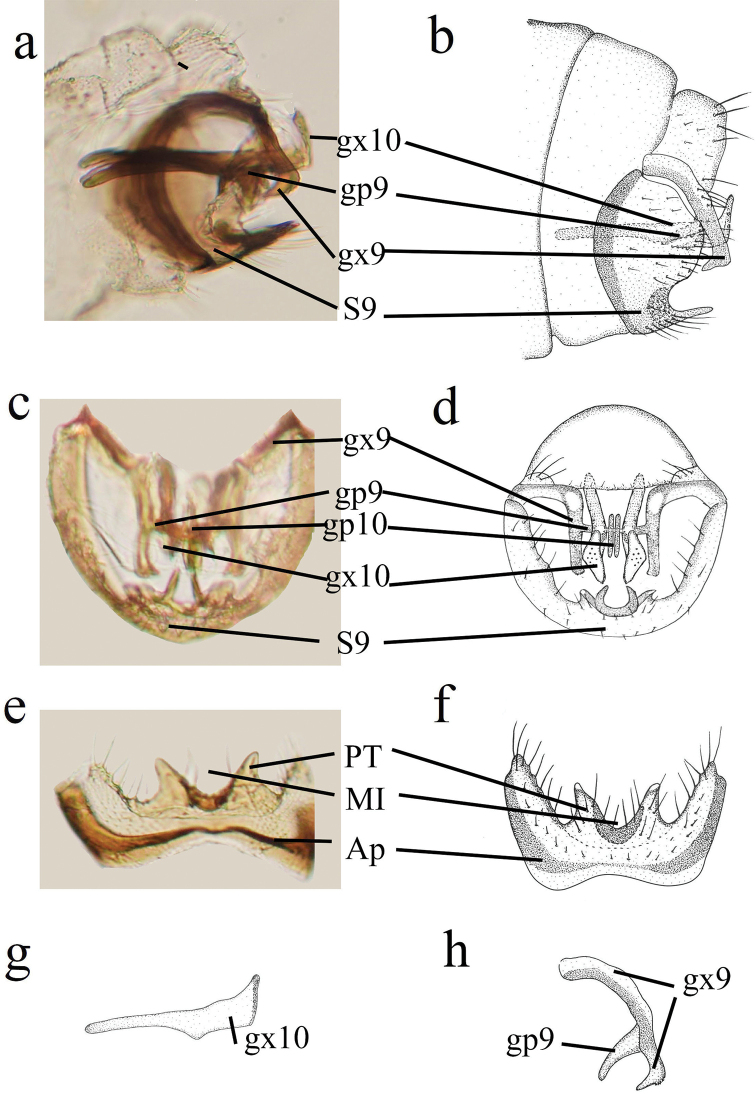
Coniopteryx (Coniopteryx) serrata sp. nov., male genitalia **a, b** genitalia, lateral view **c, d** genitalia, caudal view **e, f** sternite 9, ventral view **g** gonocoxites 10, lateral view.

***Male terminalia*** (Fig. 10a–h). Sternite 9 slightly higher than wide in lateral view; anterior margin arched in lateral view; apodeme along anterior margin wide, but interrupted or very thin ventrally; lateral process rounded and blunt; terminal process slender and acute in lateral view; median apical incision V-shaped with two short appendages in the middle. Gonocoxites 9 long and sinuated, distal section directed forwards perpendicularly and serrated. Gonocoxites 10 long and slender, bent upward distally, ventral process small. Gonapophyses 10 as a pair of long, slender rods.

##### Distribution.

China (Yunnan).

##### Etymology.

The species name is a Latin adjective referring to the minute serrations on the distal portion of gonocoxite 9.

##### Remarks.

The genitalia of the new species suggest a close relationship with Coniopteryx (Coniopteryx) wuyishana Yang & Liu, 1999. However, the two species differ in the shape of the sternite 9. The new species is characterized by having a V-shaped median apical incision while it is U-shaped in C. (C.) wuyishana. Moreover, in Coniopteryx (Coniopteryx) serrata the anterior margin of sternite 9 stretches forwards laterally and the apodeme along the anterior margin is very thin and interrupted ventrally. In contrast, C. (C.) wuyishana is characterized by a straight anterior margin of sternite 9, and a ventrally complete anterior apodeme of sternite 9.

## Supplementary Material

XML Treatment for Coniopteryx (Coniopteryx) alticola

XML Treatment for Coniopteryx (Coniopteryx) tenuisetosa

XML Treatment for Coniopteryx (Coniopteryx) serrata
